# Distribution of thermophilic endospores in a temperate estuary indicate that dispersal history structures sediment microbial communities

**DOI:** 10.1111/1462-2920.14056

**Published:** 2018-02-23

**Authors:** Emma Bell, Lynsay I. Blake, Angela Sherry, Ian M. Head, Casey R.J. Hubert

**Affiliations:** ^1^ School of Natural and Environmental Sciences Newcastle University Newcastle upon Tyne, NE1 7RU UK; ^2^ Department of Biological Sciences University of Calgary Calgary T2N 1N4, Canada; ^3^ Present address: Environmental Microbiology Laboratory Ecole polytechnique fédérale de Lausanne Lausanne, CH‐1015 Switzerland

## Abstract

Endospores of thermophilic bacteria are found in cold and temperate sediments where they persist in a dormant state. As inactive endospores that cannot grow at the low ambient temperatures, they are akin to tracer particles in cold sediments, unaffected by factors normally governing microbial biogeography (e.g., selection, drift, mutation). This makes thermophilic endospores ideal model organisms for studying microbial biogeography since their spatial distribution can be directly related to their dispersal history. To assess dispersal histories of estuarine bacteria, thermophilic endospores were enriched from sediments along a freshwater‐to‐marine transect of the River Tyne in high temperature incubations (50°C). Dispersal histories for 75 different taxa indicated that the majority of estuarine endospores were of terrestrial origin; most closely related to bacteria from warm habitats associated with industrial activity. A subset of the taxa detected were marine derived, with close relatives from hot deep marine biosphere habitats. These patterns are consistent with the sources of sediment in the River Tyne being predominantly terrestrial in origin. The results point to microbial communities in estuarine and marine sediments being structured by bi‐directional currents, terrestrial run‐off and industrial effluent as vectors of passive dispersal and immigration.

## Introduction

The presence of anaerobic thermophilic bacteria in the cold seabed was first reported for sediments from Aarhus Bay, Denmark, where despite *in situ* temperatures of 0–15°C, a thermophilic strain of *Desulfotomaculum* was found to grow by reducing sulfate when samples were heated to 60°C (Isaksen *et al*., 1994). Since this discovery, several studies have shown that thermophilic bacteria constitute an exogenous, low abundance, dormant component of microbial communities in cold marine sediments throughout the world (Hubert *et al*., 2009, 2010; de Rezende *et al*., 2013; Müller *et al*., 2014, Volpi *et al*., 2017). These misplaced thermophiles belong to the phylum *Firmicutes*, which encompasses all known endospore‐forming bacteria. Their physiology with respect to temperature (growth above 40°C; Hubert *et al*., 2009) indicates that they must be being delivered to cold sediments from an external source or sources, while spore‐formation confers a survival strategy that enables persistence at temperatures much below their growth range. Large numbers of endospores of anaerobic thermophilic bacteria are delivered to cold marine sediments each year, with 6 × 10^9^ spores m^−2^ y^−1^ reported in Aarhus Bay, representing > 10% of the total endospore population (Volpi *et al*., 2017). The detection of the same 16S rRNA gene phylotypes in both the water column and the underlying seabed in Aarhus Bay supports previous assertions that seawater acts as a vector for the dispersal of thermophilic endospores in the marine environment (de Rezende *et al*., 2013; Volpi *et al*., 2017). The same dissimilatory (bi)sulfite reductase (*dsrAB*) phylotypes of endospore‐forming, thermophilic sulfate reducing bacteria (tSRB) have also been detected in both Svalbard and Aarhus Bay sediments, despite the two locations being 3000 km apart (Hubert *et al*., 2010; de Rezende *et al*., 2013). This suggests the possible long‐distance passive dispersal of endospores of thermophilic bacteria in the ocean, potentially deriving from a common source.

Passive dispersal vectors have the potential to transport microorganisms over great distances (Galand *et al*., 2010; Yamaguchi *et al*., 2012; Müller *et al*., 2014), however, the extent to which geographic barriers contribute to changes in community composition by limiting dispersal is debated (Martiny *et al*., 2006; Eisenlord *et al*., 2012; Martiny, 2015). This is because the influence of dispersal limitation can be difficult to distinguish from the selective pressures that environmental factors exert in different habitats. Endospores of thermophilic bacteria are unable to grow in cold sediments, they remain dormant and are not subject to local selection. Their presence is, therefore, exclusively due to passive transport and the relationship between source locations and dispersal vectors. This makes them particularly useful markers for dispersal studies, since once they are deposited in an environment, even if it is unsuitable for their growth, they can remain dormant but viable for thousands (de Rezende *et al*., 2013), and possibly even millions of years (Cano and Borucki, 1995; Vreeland *et al*., 2000), thereby leaving a long‐term record of their dispersal.

To use these model organisms for biogeographic studies, it is important to consider their source habitat(s) to identify dispersal vectors and determine dispersal histories. To differentiate between possible source environments, endospores from different warm origins must be measurably distinct. A number of warm environments that are connected to the cold ocean have been proposed as potential source habitats for thermophilic endospores. These include deep oil reservoirs and mid‐ocean ridges (Hubert, *et al*., 2009). In both cases, advective flow of warm geofluids could expel microorganisms from the deep biosphere into the cold ocean, providing a transport conduit from warm to cold environments (Hubert and Judd, 2010). These considerations stem from previous studies that have considered the distribution of thermophilic spores in marine systems. More recently different estuarine systems connected to both the Atlantic Ocean and North Sea have been shown to harbour thermophilic endospores in sediments (O'Sullivan *et al*., 2015). These endospores were thought to be delivered to the estuary via ocean currents, possibly deriving from a hot subsurface environment. Estuaries are dynamic ecosystems that form the transition between the terrestrial and the marine biospheres, and can act as a sink for both terrestrial and marine derived particulate material (Sherry *et al*., 2016). These environments, therefore, offer a natural system to study the dispersal of microorganisms from different sources. Here we use thermophilic endospores as tracers of dispersal in a temperate estuary to determine whether dispersal vectors from different warm microbial habitats can be distinguished.

## Results

### Geography and sediments of the Tyne estuary

**Figure 1:**
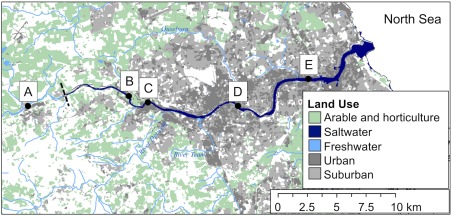
The River Tyne, a tidal estuary in the northeast of England. Sediment was collected from five stations; A, B, C, D and E. The tidal limit of the estuary is located between station A and B, indicated by the dashed line. Land use data were accessed from the EDINA Environment Digimap Service (Land Cover Map, 2007).

The River Tyne is a tidal estuary in northeast England that discharges into the North Sea. The estuary is brackish up to the tidal limit (Fig. 1) and it is estimated that 250 000 tonnes of sediment that accumulates annually in the Tyne estuary is of marine origin, while 350 000 tonnes is of terrestrial origin (Hall, 1967). The River Tyne is temperate, reaching maximum surface water temperatures of 22°C in summer months (Blake, 2010). The two principal tributaries, the North Tyne and the South Tyne, account for 90% of the total freshwater input (Spencer *et al*., 2007). The North Tyne drains areas of thick blanket peat delivering humic‐rich waters to the Tyne (Baker and Spencer, 2004, Ahad *et al*., 2008). Other sources of organic matter include consented discharges from sewage treatment works, industrial discharges and storm sewage discharges (Baker and Spencer, 2004).

Sediments from five sampling locations throughout the River Tyne were selected as inocula to test for the presence of tSRB and other spore‐forming thermophiles. These included one station upstream of the tidal limit (station A; Fig. 1) and four stations influenced by tidal activity (stations B–E; Fig. 1). Total organic carbon (TOC) contents of the sediments ranged from 0.19% ± 0.01% to 7.03% ± 0.10% (Table 1). Sediment from station A was sandy and had the lowest TOC, whereas the highest TOC was observed in stations B, C and D (Table 1).

**Table 1:** Station descriptions.

					MPN 95% Confidence limits	
Station	Latitude/longitude	TOC (weight %) (*n* = 3)	Sediment description	MPN/g sediment	Low	High
A	54°57′56″N	0.19	Sandy	2.3 × 10^1^	4.6 × 10^0^	9.4 × 10^1^
	1°52′10″W	±0.01				
B	54°58′47″N	7.03	Black mud	NM	–	–
	1°44′35″W	±0.03				
C	54°57′51″N	6.42	Black mud	4.6 × 10^3^	9.0 × 10^2^	2.0 × 10^4^
	1°40′60″W	±0.10				
D	54°58′22″N	6.42	Black mud	NM	–	–
	1°35′38″W	±0.05				
E	54°59′27″N	2.42	Sandy mud	NM	–	–
	1°28′35″W	±0.09				

NM denotes not measured. ± provides standard deviation.

### Thermophilic endospore‐forming *Firmicutes* in the Tyne estuary

**Figure 2:**
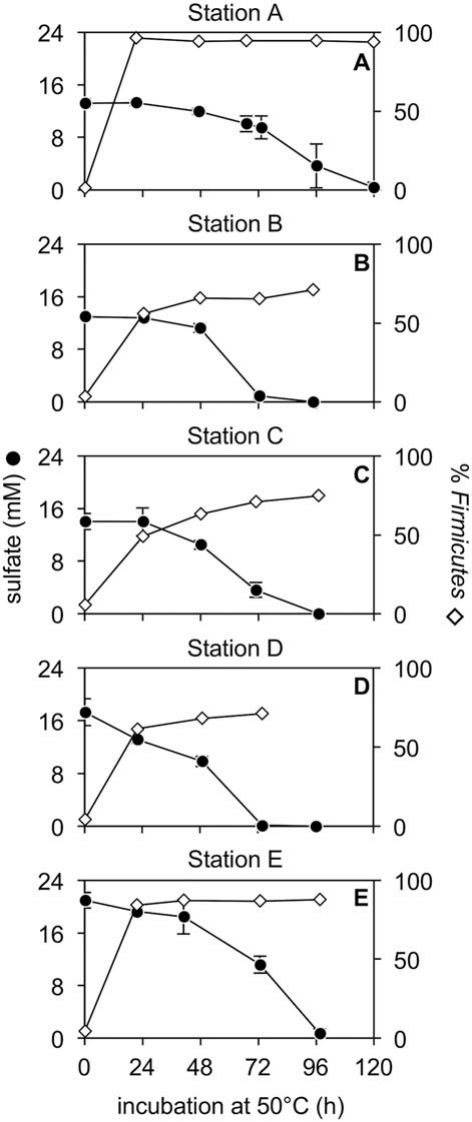
Sulfate reduction in sediment slurries amended with organic substrates and pasteurized (1 h at 80°C) prior to incubation at 50°C. Sediment slurries from non‐tidal (A) and tidally influenced sections (B–E) of the estuary were included. Error bars show standard deviation among triplicate bottles (in many cases, the error bars are smaller than the sulfate symbols). The relative abundance of the phylum *Firmicutes* (% of 12 852 reads) is shown for each sampling point.

Five sediments from the River Tyne (stations A–E) were used to prepare brackish slurries that were amended with organic substrates and pasteurized (1 h at 80°C) prior to incubation at 50°C. Sulfate reduction was detected within 24–48 h in incubation experiments for each station (Fig. 2A–E). This confirmed the presence of viable spore‐forming thermophilic sulfate‐reducing bacteria both in tidal sediments (stations B‐E; Fig. 1) and upstream of the tidal limit (station A; Fig. 1). Sulfate reduction was rapid with the sulfate supply always being exhausted within 120 h incubation at 50°C.

16S rRNA gene amplicon libraries were generated from unincubated sediments (i.e., 0 h, prior to incubation and pasteurization) and every 24 h during incubation at 50°C until sulfate was exhausted. Comparing 16S rRNA gene amplicon libraries from unincubated sediments with those from pasteurized sediments incubated at 50°C for 24 h always revealed a clear increase in *Firmicutes* (Fig. 2A–E), the phylum to which all known endospore‐forming bacteria belong. This is consistent with endospore germination and growth during incubation at 50°C.

Two of the sediments, representing freshwater (station A) and brackish (station C), were used to obtain Most Probable Number (MPN) estimates for endospores of tSRB capable of growth at 50°C. Using a three‐tube MPN approach, an abundance of 4.3 × 10^3^ spores g^−1^ sediment was estimated for brackish station C (Table 1). MPN incubations for the freshwater station A indicated a lower abundance of endospores of tSRB with only 2.3 × 10^1^ spores g^−1^ sediment (Table 1).

### Spatial distribution of endospores of thermophilic bacteria in the Tyne estuary

16S rRNA gene amplicon libraries revealed an increase in the relative abundance of 75 operational taxonomic units (OTUs) in heated sediment incubations from the River Tyne, all of which belonged to the phylum *Firmicutes*. In these sediment heating experiments, an OTU was defined as enriched if it was absent or rare (< 0.1%) in unincubated sediments (i.e., 0 h, prior to pasteurization and incubation), and increased in relative abundance (> 1%) following pasteurization and heating of the sediment to 50°C. In this way, OTUs of sulfate reducing *Desulfotomaculum* spp. were detected at every location, consistent with the observed reduction of sulfate in all sediment incubations (Fig. 2A–E). Other OTUs detected by this approach consisted of putative thermophilic fermentative microorganisms predominantly belonging to the families *Clostridiaceae* (20 OTUs)*, Tissierellaceae* (seven OTUs) and *Ruminococcaceae* (four OTUs) (Supporting Information Table S1).

**Figure 3:**
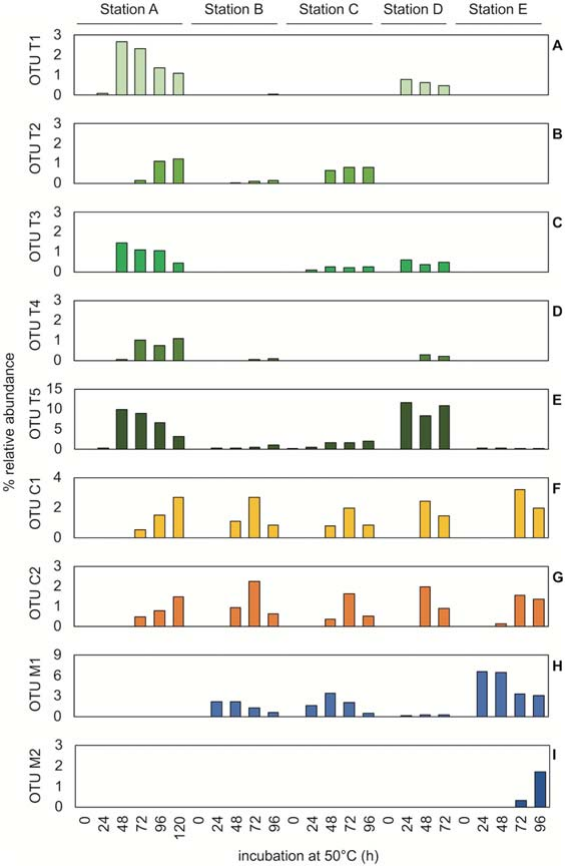
*Desulfotomaculum* OTUs enriched in pasteurized sediment heating experiments at 50°C. *Desulfotomaculum* OTUs were designated as; terrestrial (OTUs T1‐T5; A–E), cosmopolitan (OTUs C1 & C2; F, G) or marine (OTUs M1 & M2; H, I) based on their spatial distribution. All libraries were rarefied to 12 852 reads, on which relative abundance calculations (percentages) are based.

To use endospores as biogeography tracers and uncover the dispersal histories of thermophilic endospores in the Tyne estuary, distribution patterns were determined for all 75 OTUs enriched in sediment heating experiments from River Tyne (as summarized in Supporting Information Table S1, and presented for all *Desulfotomaculum* OTUs in Fig. 3). Based on the spatial distribution of enriched OTUs, they were categorized as marine or terrestrial. An OTU was classified as terrestrial if it was enriched in sediment incubations from station A, upstream of the tidal limit (e.g., Fig. 3A–G). This is consistent with delivery from an upstream, terrestrial source. Out of the 75 OTUs enriched in the Tyne estuary, 45 were enriched in sediment incubations from station A (Supporting Information Table S1; OTUs T1‐T40, C1‐C5). Downstream dispersal of these OTUs was variable; 20 of the 45 OTUs were only enriched (> 1% relative abundance) from station A (e.g., Fig. 3D).

The remaining OTUs (30/75) were only enriched in tidal sediments and were not enriched upstream of the tidal limit (e.g., Fig. 3H–I). This distribution is consistent with tidal dispersal; however, this pattern alone cannot rule out that these endospores were terrestrial in origin but were delivered to the estuary downstream of the tidal limit, e.g., via tributaries or other sources close to stations B‐E.

### Evaluating tidal currents as a dispersal vector for endospores

To further evaluate the possible marine provenance of the 30 OTUs enriched in the four different tidal sediments, marine sediment from the North Sea was sampled for additional experiments (station F). MPN enrichments, prepared using the same conditions as for station A and C, showed that station F harboured 4.6 × 10^3^ viable tSRB able to grow under brackish conditions, i.e., similar to the tidal station C (Table 1).

**Figure 4:**
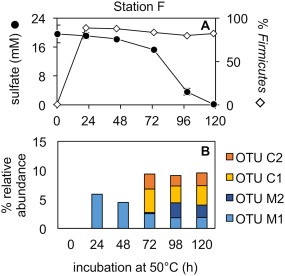
Sulfate reduction in North Sea sediment slurries (station F) amended with organic substrates and pasteurized (1 h at 80°C) prior to incubation at 50°C (A). North Sea sediment slurries were prepared with marine salinity medium. Error bars show standard deviation among triplicate bottles. The relative abundance of the phylum *Firmicutes* (% of 12 852 reads) is shown for each sampling point (A). The relative abundance of enriched *Desulfotomaculum* OTUs is shown in (B).

High temperature incubations with station F marine sediment were prepared using medium with seawater salinity to determine whether the same endospore OTUs enriched in tidal estuarine sediments were present in the North Sea, and capable of germination and growth under marine conditions. This would support their presence in the River Tyne being the result of tidal activity and dispersal into the estuary from the marine environment. In these experiments sulfate was again exhausted within 120 h of incubation and the relative abundance of *Firmicutes* increased after 24 h incubation at 50°C (Fig. 4A). This response was similar to that observed for estuarine sediment enrichments (Fig. 2A–E).

Of the 30 OTUs enriched from tidal sediments (stations B‐E) suspected to be of marine origin, nine were enriched in marine sediment incubations from station F. This included two *Desulfotomaculum* OTUs (Fig. 4B; OTUs M1 & M2). These OTUs were, therefore, classified as marine (Supporting Information Table S1; OTUs M1‐M9). Absence of estuary OTUs from marine station F experiments could be due to dispersal limitation (i.e., not reaching the sea) or inability to grow at marine salinity, or both. OTUs absent from station F were accordingly classified as terrestrial (Supporting Information Table S1; OTUs T41‐T61).

In addition to the nine OTUs identified as marine, five out of the 45 terrestrial OTUs that were identified at freshwater station A, upstream of the tidal limit, were also enriched in the marine sediment incubated at 50°C (e.g., Fig. 4B; OTUs C1 & C2). These OTUs were, therefore, classified as cosmopolitan (Supporting Information Table S1; OTUs C1‐C5) as they were found in non‐tidal, tidal and marine sediments, and exhibit a metabolic flexibility for growth under both brackish and marine conditions. Overall, of the 75 OTUs enriched in the River Tyne, 61 were classified as terrestrial, 9 as marine and 5 as cosmopolitan (Supporting Information Table S1).

### Environmental ontologies predict source habitats of estuarine thermophilic spores

**Figure 5:**
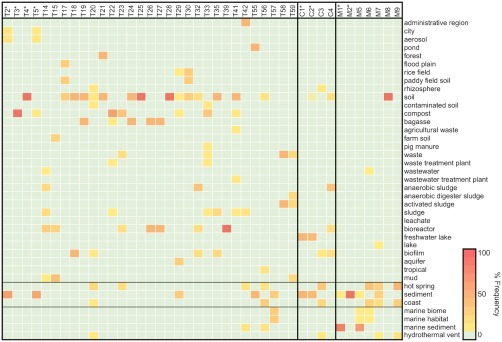
Heatmap showing the frequency of Environmental Ontology (EnvO) terms associated with *Firmicutes* OTUs enriched in 50°C incubations of pasteurized River Tyne (stations A–E) and North Sea (station F) sediment slurries. For simplicity, only a subset of the most prevalent OTUs are included, i.e., OTUs enriched in more than one library. Those excluded from this figure are presented in Supporting Information Table S2. *Desulfotomaculum* OTUs are indicated by an asterisk (*).

In addition to assessing dispersal histories for enriched *Firmicutes* based on their spatial distribution (Fig. 3 and Supporting Information Table S1), warm environments harbouring closely related bacteria to those detected in this study were identified and evaluated as potential source habitats. Potential source habitats were explored by retrieving associated environmental and contextual data for closely related sequences using the text mining software tool Seqenv (Sinclair *et al*., 2016). Of the 75 OTUs, 53 were assigned Environmental Ontology terms (EnvO) associated with closest sequence matches, as well as the frequency each OTU was associated with an EnvO term. This resulted in an environmental profile for each of the 53 OTUs (Fig. 5 and Supporting Information Table S2) e.g., *Desulfotomaculum* OTU T5 was associated with the environmental terms ‘sediment’ (62.5%), ‘aerosol’ (12.5%), ‘city’ (12.5%) and ‘compost’ (12.5%). Those OTUs (22/75) that were not assigned an environmental profile either fell below the minimum identity requirement or had no best hit associated with EnvO terms.

Environmental profiles largely supported the source assignments given to each OTU based on their spatial distribution. OTUs assigned as terrestrial based on their spatial distribution were predominantly associated with terrestrial environments and land‐based human activities, e.g., soil, compost, wastewater treatment plants (Fig. 5). Seven terrestrial OTUs included a marine habitat in their environmental profile (e.g., OTU T42; administrative region 50%, sludge 16.67%, hot spring 16.67% and marine sediment 16.67%). The environmental profiles of marine OTUs were consistent with marine and coastal habitats. Three marine OTUs had some association with the terrestrial habitats; ‘wastewater’ (OTU M6; 10%), ‘lake’ (OTU M7; 10%) and ‘soil’ (OTU M8; 100%). Cosmopolitan OTUs were associated with predominantly terrestrial and coastal habitats, as well as a ‘hydrothermal vent’ (OTU C3; 12.5%).

### Distribution of *Desulfotomaculum spp*. and habitats where closely related bacteria have been detected

**Figure 6:**
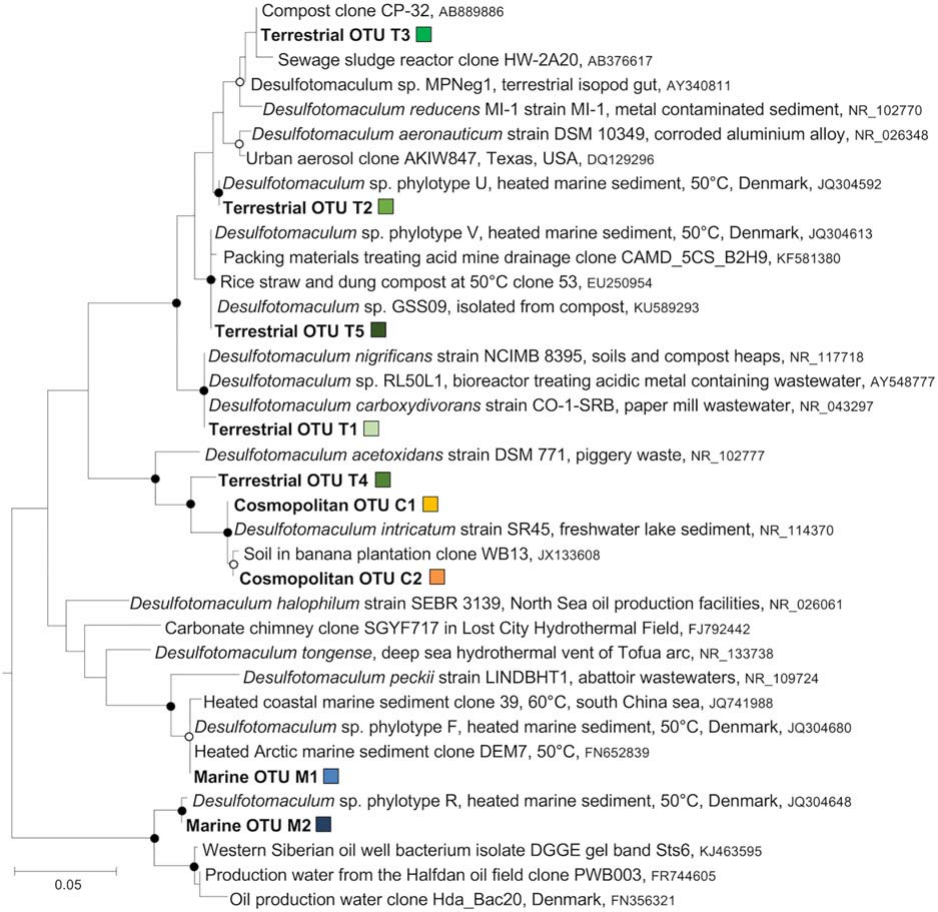
16S rRNA tree of *Desulfotomaculum* spp. with gene sequences derived from heated sediments in this study shown in bold. Coloured squares correspond to OTU colouring in Figs. 3 and 4. Closely related sequences were retrieved from Genbank and included in the phylogenetic analysis, performed using MEGA 5.2 by Maximum Likelihood with a total of 372 nucleotide positions. Filled and open circles at branching nodes indicate bootstrap support values of > 90% and 70%–90% respectively (1000 re‐samplings). The out‐group was *Helicobacteraceae* clone DVBSD (accession# KF463694).

*Desulfotomaculum* OTUs were detected in all locations investigated. Of the nine *Desulfotomaculum* OTUs, terrestrial (5), marine (2) and cosmopolitan (2) distributions were all observed (Fig. 3A–I). These OTUs were considered in more detail to distinguish dispersal vectors involving different warm source habitats. Representative 16S rRNA gene sequences (V4/V5 region) from amplicon libraries were compared with sequences from their closest relatives (Fig. 6). Closely cultured relatives (97%–99% sequence bp identity) to terrestrial OTUs include moderate thermophiles *Desulfotomaculum reducens* strain MI‐1, *Desulfotomaculum nigrificans* strain NCIMB 8395 and *Desulfotomaculum carboxydivorans* strain CO‐1SRB (Fig. 6). Each of these organisms was isolated from a terrestrial habitat; *D. reducens* from metal contaminated estuary sediments (Tebo and Obraztsova, 1998), *D. nigrificans* from soil and compost heaps (Campbell and Postgate, 1965) and *D. carboxydivorans* from paper mill effluent (Parshina *et al*., 2005) as shown in Fig. 6. All are reported to use electron donors for sulfate reduction that were included in our Tyne sediment heating experiments. Other sequences with high identities to terrestrial *Desulfotomaculum* OTUs T1‐T5) were also detected in culture‐independent evaluations of warm terrestrial environments associated with human activity such as compost and wastewater (Fig. 6; accession numbers AB889886, EU250954, KU589293, KF581380, AY548777), consistent with the environmental ontology profiles for each of these OTUs (Fig. 5) and hence indicative of dispersal from a terrestrial habitat.

Terrestrial *Desulfotomaculum* OTUs were not enriched from marine sediment from station F (max 0.01% in unrarefied libraries). Experiments from station F used marine medium so the absence of OTUs classified as terrestrial in this marine environment could indicate these bacteria are unable to grow at seawater salinity. However, OTUs T1‐T4 also displayed very low relative abundance (Fig. 3A–D and Supporting Information Table S1; max. 0.03%) in experiments in brackish basal medium with sediment from station E at the mouth of the estuary, suggesting that seaward dispersal of OTUs T1‐T4 was limited.

Two of the four *Desulfotomaculum* OTUs enriched from marine station F (OTUs M1 and M2) were assessed as having marine origins (Fig. 3H–I). *Desulfotomaculum* OTU M1 shared high sequence identity with *Desulfotomaculum* phylotypes detected in other studies with heated sediments, from the Baltic Sea (de Rezende *et al*., 2013; 99%), from Svalbard (Hubert *et al*., 2010; 99%) and from the south China Sea (Ji *et al*., 2012; 99%) (Fig. 6); the cold sediments in those locations cannot be a source habitat, hence this result points rather to broadly active forces that contribute to the dispersal of highly similar spore formers. The closest cultured relatives to OTU M1 were moderate thermophiles *Desulfotomaculum peckii* strain LINDBHT isolated from abbatoir wastewaters (Jabari *et al*., 2013; 96%) and *Desulfotomaculum tongense* strain TBGO isolated from a deep sea hydrothermal vent of the Tofua arc (Cha *et al*., 2013; 94%). *Desulfotomaculum* OTU M2 has no closely related cultured relatives (i.e., all < 92% identity), but shares high 16S rRNA gene sequence identity with uncultured *Desulfotomaculum* spp. detected in heated marine sediments from the Baltic Sea (de Rezende *et al*., 2013; 99% identity), from a Western Siberian oil well (accession KJ463595; 96% identity) and from a North Sea oil field produced water (Gittel *et al*., 2009; 95%) (Fig. 6).

Cosmopolitan OTUs C1 and C2 shared greatest sequence identity with *Desulfotomaculum intricatum* strain SR45 (99% and 100% sequence identity for OTUs C1 and C2 respectively) a moderate thermophile isolated from freshwater lake sediment in Japan (Watanabe *et al*., 2013) (Fig. 6). *Desulfotomaculum intricatum* is only able to grow in the salinity range of 0%–0.1%, underscoring how organisms closely related at the rRNA gene level can still be physiologically different, i.e., OTUs C1 and C2 were enriched in experiments with up to 2% NaCl.

## Discussion

### Thermophilic endospores delivered to the River Tyne from a terrestrial source

Thermophilic *Firmicutes* enriched in this study, appear to be a mixture of terrestrial and marine derived populations transported into the estuary via different dispersal vectors. The presence of thermophiles in sediments upstream of the tidal limit indicates that there must be terrestrial sources of thermophilic endospores. The dispersal of waterborne microorganisms upstream, past the tidal limit, could occur during storm events with high winds and high water levels resulting in the formation of wind‐blown aerosols (Aller *et al*., 2005; Crump *et al*., 2012). However, this dispersal mechanism is likely to be limited in magnitude as compared to the downstream dispersal of microorganisms in riverine flow. Therefore, endospores that were enriched upstream of the tidal limit likely come from a terrestrial source. This suggests that OTUs described as cosmopolitan are delivered to marine sediments by river discharge into the North Sea. The success of cosmopolitan OTUs in heated marine sediment microcosms (as compared to terrestrial OTUs which were not detected) may be due in part to their broader salinity tolerance permitting growth in both terrestrial and marine environments. The environmental profiles of cosmopolitan OTUs (Fig. 5) consisted of predominantly terrestrial habitats, supporting the conclusions that they come from terrestrial sources.

Terrestrial‐derived endospores (e.g., *Desulfotomaculum* OTUs T1‐T4) appear to be dispersal limited relative to OTUs C1 and C2 and other *Firmicutes* that were detected at all six locations (Supporting Information Table S1). MPN estimates indicated a relatively low abundance of tSRB spores in sediments upstream of the tidal limit. Low abundance could account for the limited dispersal downstream with decreasing numbers of endospores being deposited at increasing distances from a terrestrial inland source upstream of station A. However, sediment texture has been shown to affect endospore abundance in aquatic sediments, with significantly lower numbers found in sandy sediments compared to black muds with higher TOC (Fichtel *et al*., 2008); this is different to what is known for biogeochemically active microbial communities which can reach high abundances in sandy sediments (de Beer *et al*., 2005; Musat *et al*., 2006). Fichtel and colleagues (2008) suggested that endospores delivered from an external source, i.e., inactive dispersing propagules, are preferentially captured in muddy sediments with small pore space and lower hydraulic conductivity. Low TOC sandy sediment at station A may, therefore, retain fewer endospores as compared to other stations consisting of muddy sediments with higher TOC (Table 1) and not necessarily be reflective of the numbers of endospores carried downstream. MPN assays in this study used brackish medium, therefore, our abundance estimate only reflects endospores capable of growth in brackish conditions. Sediments from station A may retain a greater number of endospores adapted to growth at lower salinity, which were not assessed by this study.

Some terrestrial *Desulfotomaculum* OTUs had a greater relative abundance at station D (Fig. 3A and E) coinciding with a lower relative abundance of marine OTU M2 at this particular site compared to other estuary locations (Fig. 3H). Site D is at the confluence of the Tyne and the Ouseburn rivers and is downstream of the River Team and River Derwent, both of which join the Tyne downstream of station C. These three tributaries account for 10% of the freshwater discharge into the estuary (Spencer *et al*., 2007) and could transport a supply of terrestrial‐derived endospores to the Tyne estuary. This influx of freshwater discharge downstream of station C could result in the observed community composition of thermophilic *Desulfotomaculum* OTUs at station D being more similar to A than to B and C (Fig. 3).

Endospores with a terrestrial‐derived distribution pattern were related to bacteria that had been detected in paper mill wastewater, compost, domestic wastewater sludge and metal rich mine waters (Figs. 5 and 6). Within the River Tyne catchment there are a number of potential sources similar to these habitats, including discharge from industry. Hot industrial effluents are cooled before being discharged into the estuary and could carry thermophilic endospores, e.g., hot waters from a woodchip factory are cooled to 25°C before discharging near Hexham, upstream of station A. Paper mill effluent is discharged into the River South Tyne, also upstream of station A. Another industrial source could be sewage treatment works located near station E which discharges treated domestic wastewater into the River Tyne. While industrial discharges into the Tyne estuary are regulated by trade effluent consents to protect the environment (Environment Agency, 2013), discharged fluids could still contain microbial spores, resulting in their delivery to the estuary from an industrial source.

Historically, groundwater discharge from coal and metal mines and associated spoil heaps were major sources of pollution to the Tyne estuary. Water discharges from mine adits and the erosion of spoil heap material still contaminate the River Tyne, resulting in metal rich sediments within the estuary (Hudson‐Edwards *et al*., 1996). Mine water treatment schemes are in place within the estuary, such as reed beds that co‐treat mine water and sewage effluent (Johnson and Younger 2006). Treated water is discharged into the River Team, which joins the River Tyne between stations C and D. In addition, agricultural activity in the Tyne catchment could lead to diffuse pollution from agricultural run‐off sources that include soil, fertilizers and manure into the estuary. Microorganisms in compost degrade and utilize organic compounds, and the heat generated through this metabolism can maintain the temperatures up to 70°C (Maeda *et al*., 2010). During compost processing, thermophilic microorganisms can be released into the air, which then acts as a dispersal vector that deposits cells in nearby temperate soils (Thummes *et al*., 2007). The mature compost product is ultimately returned to the land as organic fertilizer, which may contain spores of thermophilic bacteria that could subsequently wash into the river catchment.

### Marine thermophilic endospores seeding the Tyne estuary

Some OTUs have a distribution pattern suggestive of a marine source environment (Fig. 3H and I). Marine OTUs were enriched in estuarine sediments, primarily near the mouth of the estuary (station E) and to a lesser extent at upstream locations B‐D, indicating that these organisms may be coming from the North Sea and dispersed several kilometres upstream into the estuary. In contrast to downstream dispersal in riverine flow for other OTUs, the distribution pattern observed for marine OTUs is consistent with tidal currents seeding estuarine sediments with marine derived thermophilic endospores.

Warm deep biosphere environments such as petroleum‐bearing sediments and mid‐ocean ridge habitats have been proposed as sources of thermophilic endospores deposited in cold marine surface sediments (Hubert *et al*., 2009, 2010) and are both plausible origins of marine‐derived thermophiles detected in the Tyne estuary. Modern day oil industry activity in the North Sea could contribute to the dispersal of thermophilic endospores from the subsurface via discharged oil field production waters – one of the largest waste products routinely released into seawater (Yeung *et al*., 2011). This discharge can transport significant numbers of microorganisms from the subsurface into the ocean, with an estimated 3–16 kg of cells expelled daily in production fluids from high temperature (70–110°C) oil reservoirs in the North Sea alone (Parkes and Sass, 2009). In recent decades, the amount of produced water discharged into the North Sea has significantly increased owing to extension of offshore activities. As oil reservoirs reach later production phases larger fractions of water are co‐produced with oil during secondary recovery operations. The maturation of many producing oil fields in the North Sea is resulting in larger volumes of water both being injected for secondary oil recovery and co‐produced during oil extraction (OSPAR Commission, 2000). Produced water discharge could thus be an anthropogenic large‐scale dispersal vector for transporting subsurface bacteria into the ocean, since many North Sea oil fields are hot and anoxic and known to harbour anaerobic thermophilic *Firmicutes* (Rosnes *et al*., 1991; Nilsen *et al*., 1996; Gittel *et al*., 2009; Vigneron *et al*., 2017).

In addition to produced water, natural seepage of hydrocarbons is estimated to account for 200 000–2 000 000 tonnes of crude oil entering the oceans globally each year (National Research Council, 2002) and could similarly be a source of subsurface derived microorganisms (Hubert and Judd, 2010). *Desulfotomaculum* OTU M2 was detected in greatest relative abundance in North Sea (station F) sediments and was related to 16S rRNA gene amplicons from North Sea oil field produced water (Fig. 6; Gittel *et al*., 2009). Given the proximity and magnitude, hot North Sea oil fields, whether via natural seepage or production water discharge, are a likely source of thermophilic microorganisms in North Sea marine and tidal sediments.

### Thermophilic endospores as microbial indicators of pollution

A study conducted near a discharge site at a Canadian offshore oil platform in the northwest Atlantic Ocean found that produced water had a detectable effect on the sediment bacterial community within 250 m of the discharge site, but that more distant sediments and surrounding seawater had a stable bacterial community which did not appear to be affected by the produced water (Yeung *et al*., 2011). In a later study of the same platform, an anaerobic thermophilic endospore‐former (*Thermoanaerobacter* sp.) found in the produced water was targeted as a signature microorganism to monitor dispersion from the discharge site (Yeung *et al*., 2015). *Thermoanaerobacter* sp. was subsequently detected up to 1 km from the oil platform using q‐PCR and nested‐PCR. It is not clear whether the methods used in these Atlantic Canada studies are capable of extracting DNA from spores, and it is possible that beyond 1 km only spores are further dispersed and thus no longer detectable by DNA extraction and PCR‐based molecular methods. In our study, thermophiles were not readily detected in sediments without incubation at high temperature. This has been observed in other sediment heating experiments (Hubert *et al*., 2009; de Rezende *et al*., 2013) and in experiments with seawater, where thermophilic *Desulfotomaculum* were detected in the water column only following incubation at a suitable growth temperature (de Rezende *et al*., 2013). Sediment heating experiments that promote endospore germination may, therefore, reveal a greater impact area of produced water discharge in the marine environment than was observed by Yeung *et al*., (2015), and could contribute to future studies assessing changes in the microbial community as an indicator of pollution.

The use of microbial indicators for pollution is already widely applied in water quality assessments, in particular for the detection of pathogens (Tallon *et al*., 2005). Microbial indicators used for water quality also rely on endospore germination for detection, e.g., the spore‐former *Clostridium perfringens* is used as an indicator of faecal pollution and its detection relies on culture‐based methods that allow for germination and growth before enumeration (Vierheilig *et al*., 2013). In a similar way, thermophilic microorganisms may be used as part of petroleum exploration strategies as indicators of hydrocarbon seeps (Hubert and Judd, 2010). Thermophilic spore‐forming bacteria represent particularly good targets for microbial exploration strategies, as they are conspicuous in cold sediments and are readily detectable using high temperature incubation assays. However, the true subsurface provenance of putatively reservoir derived thermophiles must be carefully assessed given the multitude of source environments and dispersal vectors that can be uncovered, as revealed by this study of the Tyne estuary (e.g., Fig. 5). Discoveries of thermophilic endospores associated with industrial activities in this study presents the possible application of using spore‐formers as indicators of pollution from multiple industrial processes that discharge effluent into surface water environments of interest, e.g., in highly populated municipal areas such as those surrounding the Tyne estuary. Characterisation of effluents related to specific activities to identify distinct target microorganisms could be used as a strategy to track the dispersion of pollutants in the aquatic environment. Candidate microorganisms for this kind of application could be endospores which appear to be more restricted in their distribution, e.g., OTUs belonging to the families *Tissierellaceae*, *Ruminococcaceae*, *Symbiobacteriaceae* and *Lachnospiraceae* (Supporting Information Table S1) that were only found in freshwater and brackish sediments. Endospore longevity in the environment means that they can also be used as indicators of long‐term or historic pollution, or past environmental conditions, when endospore populations are studied as a function of depth (Wunderlin *et al*., 2014).

### Conclusion

The results presented here show that the distribution of spore‐forming thermophiles can be used to explain the passive dispersal of microorganisms in aquatic environments. The distribution of thermophilic *Firmicutes* in the River Tyne indicates that while tidal currents do seed estuarine sediments with marine microorganisms, the greater proportion of OTUs detected (88%) are apparently derived from terrestrial environments. This proportion is consistent with sources of sediment to the estuary being predominantly terrestrial (58%; Hall, 1967) and suggests that the majority of microorganisms introduced to estuarine sediments are delivered via terrestrial run‐off, groundwater discharge and effluent from industrial activities. The dispersal of microorganisms downstream and into the sea is apparently limited for many terrestrial derived micro‐organisms, however, so‐called cosmopolitan microorganisms represented 16%–30% of the enriched community in marine sediment (Supporting Information Table S1). This suggests that microorganisms with a terrestrial source are both delivered to marine sediments and are able to compete with marine microorganisms given the appropriate growth conditions. The passive dispersal of microorganisms into and out of the estuary in this way may contribute to the microbial seed bank in estuarine and marine sediments. Microorganisms from the seed bank may be capable of responding to environmental change, which has important implications for ecological processes which influence microbial diversity, such as succession and recovery following disturbance events (Fierer and Lennon, 2011; Caporaso *et al*., 2012a; Gibbons *et al*., 2013). The exchange of microorganisms observed here between the terrestrial and marine biospheres may, therefore, contribute to the maintenance of microbial diversity in aquatic environments.

## Experimental procedures

### Sediment sampling

Sediment was collected from six stations (Fig. 1); Station A (54°57′56″N, 1°52′10″W), Station B (54°58′47″N, 1°44′35″W), Station C (54°57′51″N, 1°40′60″W), Station D (54°58′22″N, 1°35′38″W), Station E (54°59′27″N, 1°28′35″W) and Station F (55°05′13″N, 1°15′09″W). Station A is upstream of the tidal limit of the estuary, where the river is tree‐lined and adjacent to agricultural land. Stations B‐E are within the tidal range, where the river is channelized and flows through the urbanized city of Newcastle upon Tyne. Station F is ca. 175 km off the English northeast coast, where the River Tyne discharges.

Sediment from stations B and C were collected at low tide with a trowel. Light brown oxidized surface sediments (∼0–2 cm) were removed and deeper black anoxic sediment was collected down to ∼20 cm depth with a trowel. Sediment from station A was collected in the same way, but there was no apparent distinction between oxidized and anoxic sediment. Marine sediment and estuarine sediments from the navigable part of the estuary and North Sea (stations D–F) were collected from aboard the *RV Princess Royal* with a Van Veen grab sampler. Sulfate is present in the sediment porewater of tidally influenced sediments (e.g., 6 mM at station B, the tidal station that is the farthest upstream; Blake, 2010). All sediments were stored in sealed containers at 4°C. Sediment descriptions were determined following visual inspection (Table 1).

### Sediment slurry incubations at elevated temperature

Sediments from freshwater (A) and brackish stations (B‐E) were mixed with brackish basal medium prepared according to Widdel and Bak (1992) with the concentration of sulfate adjusted to 20 mM. The medium was amended with tryptic soy broth to a final concentration of 3 g l^−1^, glucose at a final concentration of 3 mM, and the organic acids acetate, propionate, butyrate and lactate at final concentrations of 3 mM each. Sediment was added to each serum bottle under a constant flow of N_2_ to give a final medium:sediment ratio of 2:1 (v/w) and the bottles were sealed using butyl rubber stoppers and aluminium crimps. Marine sediment (station F) was mixed with marine basal medium (Widdel and Bak, 1992) with the same levels of sulfate and organic substrates as indicated above. Microcosms were always prepared in triplicate from all sample locations. Microcosms were pasteurized for 1 h at 80°C to kill vegetative cells and to select for endospore‐forming bacteria. Following pasteurization microcosms were incubated at 50°C for 8 days.

### Monitoring of time course incubations

Sediment microcosms were subsampled daily by removing 2 ml of homogenized slurry from the microcosms using a N_2_ flushed syringe. Aliquots of sediment slurry were centrifuged (13 000 g, 5 min, Hettich Mikro 200). The supernatant was used for sulfate analysis and the sediment pellet was stored at −20°C for DNA extraction. Sulfate was analysed by ion chromatography using a Dionex ICS‐1000 with an AS40 auto‐sampler and an IonPac AS14A analytical column with the flow rate set to 1 ml min^−1^. The eluent was 8.0 mM Na_2_CO_3_/1.0 mM NaHCO_3_ solution and the injection loop was 25 µl. Chromatograms were visualized using Chromeleon Dionex software and peak area calibration used standard solutions of Na_2_SO_4_.

### Preparation of 16S rRNA gene amplicon libraries

DNA was extracted using the PowerSoil DNA isolation Kit (MoBio Laboratories) following the manufacturer's protocol, except for the elution step (step 20), which was modified by eluting DNA with 50 μl instead of 100 μl of elution buffer (solution C6) and allowing 30 min for elution instead of centrifuging immediately (step 21). Extracted DNA was used as a template for two‐step nested PCR and PCR products were screened by denaturing gradient gel electrophoresis (DGGE) (Supporting Information Fig. S1). Details of the PCR‐DGGE protocol are provided in the Supporting Information. The same DNA extracts were also used as a template for PCR amplification using Golay barcoded primers that target the V4‐V5 region (position 515–926) of the 16S rRNA gene (Caporaso *et al*., 2012b), specific details of which are provided in the Supporting Information. Sulfate concentrations (Figs. 2 and 4) and DGGE (Supporting Information Fig. S1) were highly reproducible, therefore, amplicons from triplicate microcosms were pooled in equimolar concentrations following quantification with the Quant‐iT Picogreen dsDNA Assay kit (Invitrogen, UK). Pooled amplicons were then purified using Agencourt AMPure XP paramagnetic beads (Beckman Coulter Life Sciences, USA). Following purifications, amplicons were quantified using the Qubit 3.0 Fluorometer and Qubit dsDNA High Sensitivity Assay kit (Invitrogen) to enable equimolar pooling from each sample to be sequenced (100 pM each). Pooled amplicons were submitted for sequencing using an in‐house Ion Torrent PGM (School of Civil Engineering and Geosciences, Newcastle University) and standard Ion Torrent sequencing procedures (Life Technologies, Inc).

### Analysis of 16S rRNA gene amplicon libraries

The Quantitative Insights Into Microbial Ecology (QIIME) software package (version 1.7.0) was used to process raw sequence data (Caporaso *et al*., 2010a). Sequences were assigned to samples based on their barcodes and simultaneously filtered to remove poor quality reads (those with a quality value of < 20 were discarded). Organisation of good quality reads into operational taxonomic units (OTUs) was performed using UClust (Edgar, 2010), with an OTU threshold defined at 0.97 (97% sequence identity). Clustering of OTUs was performed open reference against the Greengenes 16S rRNA database (DeSantis *et al*., 2006). Taxonomy was assigned using RDP Classifier (Wang *et al*., 2007) and sequences aligned using PyNAST (Caporaso *et al*., 2010b). Chimeric sequences were identified with ChimeraSlayer (Haas *et al*., 2011) and removed from subsequent analysis. Each library was rarefied to the size of the smallest library in the data set (12 852 reads) and these used to determine distribution patterns between the different samples. In certain instances, if an OTU of interest was absent in the rarefied amplicon library, the unrarefied amplicon library was checked to confirm the absence of that OTU. Mean, median and maximum library sizes were 25 729, 23 647 and 91 369 reads respectively.

Environmental Ontology terms (EnvO) were associated to select OTUs of interest using the tool Seqenv (Sinclair *et al*., 2016). Sequences were compared to the nucleotide (nt) database provided by NCBI using the BLAST algorithm (Altschul *et al*., 1990). Best hits were selected using default parameters as described by Sinclair *et al*., (2016). Of 75 OTUs entered, 53 were assigned environmental profiles consisting of a set of EnvO terms and their associated frequencies. Closest sequence matches to *Desulfotomaculum* OTUs were identified within the Genbank database using the blast (blastn) suite at the National Center for Biotechnology Information (NCBI) (Altschul *et al*., 1990). 16S rRNA amplicon sequences were aligned using BioEdit Sequence Alignment Editor 7.0.9.0 (Hall, 1999). Phylogenetic analysis was performed in MEGA 6.0 (Tamura *et al*., 2013) by Maximum Likelihood based on the Tamura‐Nei model (Tamura and Nei, 1993). Sequencing data are archived at the NCBI Sequence Read Archive under Bioproject accession PRJNA362720.

### MPN quantification of endospores of thermophilic sulfate‐reducing bacteria

Endospores of thermophilic sulfate‐reducing bacteria in sediments from stations A, C and F were enumerated using a three‐tube MPN approach. Brackish medium was used for all MPN assays to target the same endospores at all locations, and to not preferentially select based on salinity growth ranges. Brackish medium was prepared as described above and amended with the organic acids butyrate, propionate, lactate and acetate, to final concentrations of 3 mM each. Medium was then dispensed into Hungate tubes under a constant flow of N_2_. A 1:10 dilution (w/v) of sediment and medium was pasteurized for 1 h at 80°C after which the 10^−1^ dilution of sediment was serially diluted (1 ml into 9 ml medium) up to 10^−7^. Inoculated Hungate tubes as well as nine sterile blanks (containing only medium and substrates) were incubated at 50°C for three months, after which the concentration of sulfide was determined spectrophotometrically as a colloidal solution of copper sulfide as described by Cord‐Ruwisch (1985). Hungate tubes that showed sulfide production were scored positive for growth.

### Total organic carbon content

Sediment TOC was determined according to ISO 10694 (1995). The TOC of triplicate samples was analysed using a LECO CS230 carbon analyser (LECO Instrument UK) and reported as a percentage of total mass.

## Conflict of Interest

The authors declare no conflict of interest.

## Supporting information

Additional Supporting Information may be found in the online version of this article at the publisher's web‐site:

**Fig. S1.** *Desulfotomaculum* specific DGGE following two‐step nested PCR; DEM116f/1164r (targeting *Desulfotomaculum* spp.) followed by 341f‐gc/907r (universal bacterial 16S rRNA primers). Sediments were pasteurized sediments (1 h at 80°C) and incubated at 50°C. DNA was extracted after 72 h incubation. Average % similarity between triplicates was calculated in Bionumerics software package (Applied Maths, Austin, TX, USA). Band matching data were used to calculate Dice similarity indices.

**Table S1**. Relative abundance (percentage of 12 852 reads), source assignment, taxonomy and closest relatives of 16S rRNA gene sequences from OTUs enriched in pasteurized sediment slurries incubated at 50°C.

**Table S2.** Heatmap showing the frequency of Environmental Ontology (EnvO) terms associated with *Firmicutes* OTUs enriched in 50°C incubations of pasteurized River Tyne (stations A–E) and North Sea (station F) sediment slurries.
