# Supplementary figures and images for: Distribution of thermophilic endospores in a temperate estuary indicate that dispersal history structures sediment microbial communities
Source: Environ Microbiol. 2018 Feb 23;20(3):1134–47. doi: 10.1111/1462-2920.14056 (PMC6849807; doi:10.1111/1462-2920.14056)

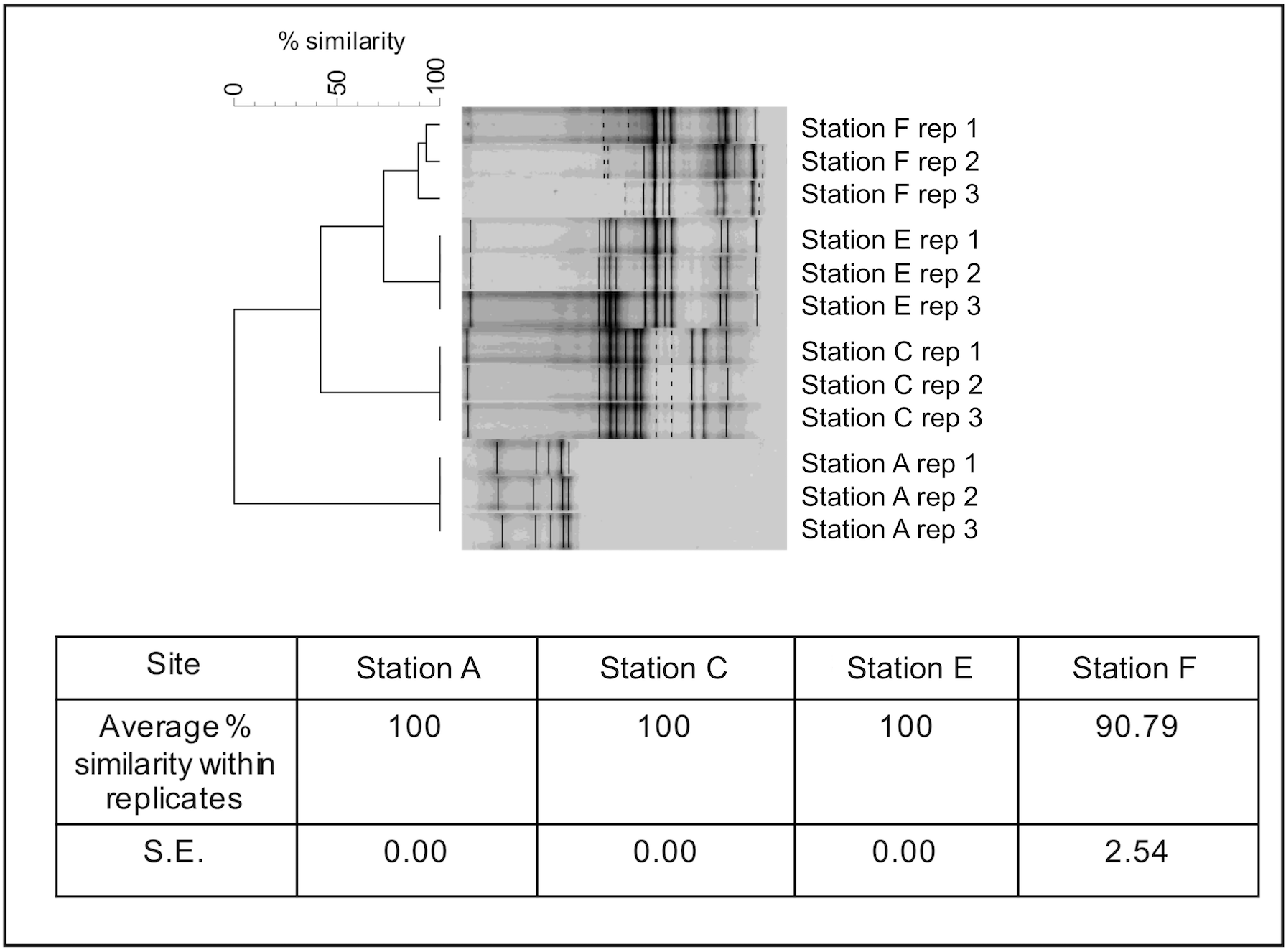

Supplement: Supplementary file 1 — Fig. S1. Desulfotomaculum specific DGGE following two‐step nested PCR; DEM116f/1164r (targeting Desulfotomaculum spp.) followed by 341f‐gc/907r (universal bacterial 16S rRNA primers). Sediments were pasteurized sediments (1 h at 80°C) and incubated at 50°C. DNA was extracted after 72 h incubation. Average % similarity between triplicates was calculated in Bionumerics software package (Applied Maths, Austin, TX, USA). Band matching data were used to calculate Dice similarity indices. [file EMI-20-1134-s001.tiff]
